# A selective defect in Fas-mediated apoptosis in HAM/TSP: An ex vivo, in vitro and in silico study

**DOI:** 10.1186/1742-4690-11-S1-P78

**Published:** 2014-01-07

**Authors:** Soraya Maria Menezes, Daniele Decanine, Ricardo Khouri, Saul Velloso Schnitman, Ramon A Kruschewsky, Anne-Mieke Vandamme, Bernardo Galvão-Castro, Johan Van Weyenbergh

**Affiliations:** 1 Clinical and Epidemiological Virology, Rega Institute for Medical Research, Department of Microbiology and Immunology, University of Leuven, Leuven, Belgium; 2 LIMI, LASP, Gonçalo Moniz Research Center (CPqGM), Oswaldo Cruz Foundation (FIOCRUZ), Salvador-Bahia, Brazil; 3 Bahiana School of Medicine and Public Health, Salvador-Bahia, Brazil; 4 Centro de Malária e outras Doenças Tropicais and Unidade de Microbiologia, Instituto de Higiene e Medicina Tropical, Universidade Nova de Lisboa, Portugal; 5 Institute for Immunological Investigation (iii-INCT), São Paulo, Brazil

## 

Fas/FasL-mediated apoptosis is crucial for a functional immune response, with deficient Fas/FasL expression being linked to several autoimmune diseases. In addition, a FAS-670 polymorphism in an interferon (IFN)-regulated STAT1-binding site has been associated to both ATL (Farre et al., 2008) and HAM/TSP (Vallinotto et al., 2012) susceptibility. Recently, Fas has also been identified as part of an IFN-regulated gene signature in HAM/TSP (Tattermusch et al., 2012). Therefore, we examined Fas expression and function in lymphocyte activation, apoptosis, lymphoproliferation and gene expression profiling, using polychromatic flow cytometry, thymidine incorporation and microarray analysis (Affymetrix) respectively, in PBMCs from 20 HAM/TSP patients, 30 asymptomatic HTLV-1-infected individuals and 34 healthy controls (Salvador-Bahia, Brazil). Fas expression was increased in both asymptomatic HTLV-1-infected individuals and HAM/TSP patients, as compared to uninfected controls. In HAM/TSP, Fas expression correlated positively to lymphocyte activation markers (HLA-DR, CD86), but negatively to disease duration, suggesting that Fas-mediated pro-apoptotic capacity decreases over time in patients. Likewise, increased Fas expression in HAM/TSP did not lead to increased apoptosis upon in vitro culture. However, in HAM/TSP patients, IFN-alpha-induced Fas expression was paralleled by decreased lymphoproliferation, suggesting a link between defective Fas-mediated apoptosis and excessive lymphoproliferation, a hallmark of HTLV-1 infection, which can be restored by IFN-alpha treatment. Taken together, our results suggest defective Fas-mediated apoptosis might be an additional factor of HAM/TSP pathogenesis, besides its recently identified IFN gene signature.

